# Physiological response and resilience of early life-stage Eastern oysters (*Crassostrea virginica*) to past, present and future ocean acidification

**DOI:** 10.1093/conphys/cou004

**Published:** 2014-03-04

**Authors:** Christopher J. Gobler, Stephanie C. Talmage

**Affiliations:** Stony Brook University, School of Marine and Atmospheric Sciences, 239 Montauk Highway, Southampton, NY 11968, USA

**Keywords:** Bivalve, ocean acidification, oyster, restoration

## Abstract

The Eastern oyster, *Crassostrea virginica* (Gmelin, 1791), is the second most valuable bivalve fishery in the USA and is sensitive to high levels of partial pressure of CO_2_ (pCO_2_). Here we present experiments that comprehensively examined how the ocean's past, present and projected (21st and 22nd centuries) CO_2_ concentrations impact the growth and physiology of larval stages of *C. virginica. Crassostrea virginica* larvae grown in present-day pCO_2_ concentrations (380 μatm) displayed higher growth and survival than individuals grown at both lower (250 μatm) and higher pCO_2_ levels (750 and 1500 μatm). *Crassostrea virginica* larvae manifested calcification rates, sizes, shell thicknesses, metamorphosis, RNA:DNA ratios and lipid contents that paralleled trends in survival, with maximal values for larvae grown at 380 μatm pCO_2_ and reduced performance in higher and lower pCO_2_ levels. While some physiological differences among oysters could be attributed to CO_2_-induced changes in size or calcification rates, the RNA:DNA ratios at ambient pCO_2_ levels were elevated, independent of these factors. Likewise, the lipid contents of individuals exposed to high pCO_2_ levels were depressed even when differences in calcification rates were considered. These findings reveal the cascading, interdependent impact that high CO_2_ can have on oyster physiology. *Crassostrea virginica* larvae are significantly more resistant to elevated pCO_2_ than other North Atlantic bivalves, such as *Mercenaria mercenaria* and *Argopecten irradians*, a finding that may be related to the biogeography and/or evolutionary history of these species and may have important implications for future bivalve restoration and aquaculture efforts.

## Abstract (precis)

The Eastern oyster, Crassostrea virginica, is the second most valuable bivalve fishery in the US. C. virginica larvae were significantly more resistant to ocean acidification than other North Atlantic bivalves, suggesting it may be a better target for future restoration and aquaculture efforts.

## Introduction

Rising levels of atmospheric CO_2_ have led to world's oceans acidifying since the dawn of the industrial revolution, a process expected to continue for at least two centuries (Caldeira and Wickett, 2003). This ocean acidification depresses the availability of carbonate ions (CO_3_^2−^) and thus is expected to have adverse effects on a diverse assemblage of marine animals that incorporate calcium carbonate into their hard parts or shells (Orr *et al.*, 2005; Doney *et al.*, 2009). The shallow nature of coastal zones makes them more vulnerable to external fluxes of CO_2_ than open ocean regions (Duarte *et al.*, 2013). Many coastal ecosystems already experience elevated levels of CO_2_ (Salisbury *et al.*, 2008; Cai *et al.*, 2011; Hu and Cai, 2013), in part due to decomposition of the large amount of terrestrial, algal and anthropogenic organic carbon delivered to estuaries (Gattuso *et al.*, 1998; Paerl *et al.*, 1998; Thomas *et al.*, 2004; Gupta *et al.*, 2009). Furthermore, coastal zones may be impacted by discharge of acidic river water (Salisbury *et al.*, 2008; Hu and Cai, 2013) or upwelling of acidified deep waters (Feely *et al.*, 2008). Hence, animals in coastal zones are, in some cases, already subjected to acidification.

Oysters are a keystone species in coastal zones due to their economic value and the array of ecosystem services they provide, including robust water filtration and the creation of reefs that represent important benthic habitat (Newell, 2004). Many species of oyster are vulnerable to high CO_2_ levels. Larvae of the Pacific oyster (*Crassostrea gigas*) exposed to experimentally elevated CO_2_ levels experienced slowed developmental rates, slowed shell mineralization rates and malformations (Kurihara *et al.*, 2007), while juveniles have displayed significant declines in calcification with increases in partial pressure of CO_2_ (pCO_2_) to levels expected at the end of the century (Gazeau *et al.*, 2007). In contrast, decreases (−0.35 pH units) in pH did not affect the fertilization rate or sperm motility of *C. gigas* (Havenhand and Schlegel, 2009)*.* The survival of *C. gigas* larvae at a hatchery was shown to be strongly dependent on the aragonite saturation state of waters in which larval oysters were spawned and reared for the first 48 h of life (Barton *et al.*, 2012), probably due to the strong dependence of these larvae on external, rather than internal, sources of inorganic carbon for shell synthesis during this developmental period (Waldbusser *et al.*, 2013). Exposure of *C. gigas* and a second species of oyster (*Saccostrea glomerata*) to high CO_2_ and increased temperature caused declines in fertilization success, development of embryos and the size of larvae, as well as abnormal larval morphology (Parker *et al.*, 2010). Estuarine acidification has been shown to cause mortality of rock oysters (*Saccostrea glomerata*; Dove and Sammut, 2007). Pearl oysters (*Pinctada fucata*) grown in acidic seawater have weaker, malformed shells compared with normal oysters (Welladsen *et al.*, 2010). In contrast, growth and calcification of the larvae from the Suminoe oyster (*Crassostrea ariakensis*) were unaffected by pCO_2_ levels ranging from 280 to 800 μatm (Miller *et al.*, 2009). However, high levels of CO_2_ have been shown to have deleterious consequences for the Eastern oyster (*Crassostrea virginica*, Gmelin, 1791; see below).

The Eastern oyster (*Crassostrea virginica*) has a geographical range of 8000 km from Brazil to Canada in the Western Atlantic Ocean, where it inhabits a wide range of salinities (5–30) in coastal zones (Carriker and Gaffney, 1996). In the USA, *C. virginica* is the second most valuable bivalve fishery (NMFS, 2010). Waldbusser *et al.* (2011) reported that calcification rates of juvenile *C. virginica* declined significantly during experimental reductions in pH that matched those observed in Chesapeake Bay in recent decades, while Talmage and Gobler (2011) found that juvenile Eastern oysters exposed to high CO_2_ levels (∼1700 μatm) grew 40% slower than those exposed to normal CO_2_ levels. With increasing CO_2_ levels, *C. virginica* larvae experience a reduction in calcium content (Miller *et al.*, 2009) as well as decreased size and survivorship (Talmage and Gobler, 2009). Moreover, Miller *et al.* (2009) reported that *C. virginica* larvae grown with low CO_2_ levels (280 μatm) yielded slight increases in shell area and calcium carbonate (CaCO_3_) composition, suggesting that, as was the case for other bivalve species (*Mercenaria mercenaria* and *Argopecten irradians*; Talmage and Gobler, 2010), *C. virginica* larvae may experience optimal growth and performance at the CO_2_ levels that were present during the pre-industrial era.

The goal of this study was to provide a comprehensive assessment of how past, present and projected CO_2_ levels (∼250, 380, 750 and 1500 μatm pCO_2_) affect the physiology of larval Eastern oysters, *C. virginica*. Reductions in the growth and survival at this stage can cause substantial declines in adult populations (Caley *et al.*, 1996; Schneider *et al.*, 2003). Oyster larvae were exposed to each pCO_2_ level for the entirety of the larval stage, and the calcification rate, RNA:DNA ratio, shell length, shell thickness, lipid content and shell morphology were quantified. Next, we compared differences in these physiological parameters as a function of size and calcification rates of larval oysters. A final goal was to compare the vulnerabilities of *C. virginica* to elevated pCO_2_ levels with those of other North American resource bivalves. This multi-faceted approach provided a comprehensive assessment of the biological response of early life stages of this species to varying levels of pCO_2_.

## Materials and methods

### Carbon dioxide treatments and measurements

This study examined the effects of multiple pCO_2_ levels on larval stages of *C. virginica*. For all experiments, experimental vessels with bivalves (described below) were maintained in water baths at 24°C using commercially available aquarium heaters (Aquatic Eco-systems, Inc., Apopka, FL, USA), a temperature ideal for the growth and survival of *C. virginica* larvae (Matthiessen, 2001). Temperatures were recorded every 6 min throughout experiments using *in situ* data loggers (Onset^©^, Bourne, MA, USA) and remained within ±0.5°C of target values. A gas proportionator system (Cole Parmer^®^ Flowmeter system, multitube frame, Cole Parmer, Court Vernon Hills, IL, USA) was used to deliver CO_2_ gas to seawater treatments at multiple rates. The gas proportionator delivered pre-mixed gases (250, 380, 750 or 1500 μatm; Praxair^©^, Danbury, CT, USA) at flow rates which turned over the volume of experimental vessels several times an hour, ensuring that vessels did not equilibrate with the atmosphere. For experiments, the CO_2_ gas mixtures were continuously delivered to the bottom of replicated (*n * = 4) experimental vessels (detailed below). With continuous bubbling, all treatment carboys remained saturated with respect to dissolved oxygen (∼8 mg l^−1^). To quantify precise CO_2_ levels attained in experimental treatments, aliquots were removed immediately before commencing experiments as well as immediately after the conclusion of experiments and analysed immediately using an EGM-4 Environmental Gas Analyzer^®^ (PP Systems) system that quantified total dissolved inorganic carbon (DIC) levels after separating the gas phase from seawater using a Liqui-Cel^®^ Membrane (Membrana). These analyses provided a methodological precision of ±2.6% for replicated measurements of total DIC and provided full recovery (102 ± 3%) of Dr Andrew Dickson's (University of California San Diego, Scripps Institution of Oceanography) certified reference material for total inorganic carbon in seawater [Batch 102 = 2013 μmol total DIC (kg seawater)^−1^]. Levels of CO_2_ were calculated based on measured levels of total inorganic carbon, pH [mol (kg seawater)^−1^; NBS scale], temperature, salinity, and first and second dissociation constants of carbonic acid in seawater according to Roy *et al.* (1993) using the program CO2SYS (http://cdiac.ornl.gov/ftp/co2sys/). Daily measurements of pH (Thermo Scientific Orion Star Series™ Benchtop pH meter; ±0.001; calibrated prior to each use with NIST traceable standards) indicated that experimental vessels maintained a constant pH level throughout experiments (<0.5% relative standard deviation within treatments). Spectrophotometric measurements of pH made using *m*-cresol purple as described by Dickson *et al.* (2007) and corrected for scale (Dickson, 1993) were highly similar to and never significantly different from those obtained with the pH electrode. The levels of precision for measurements of pH and DIC adequately resolved the hundreds of microatmosphere differences among our CO_2_ treatments (∼250, 380, 750 and 1500 μatm).

### Larvae and treatments

*Crassostrea virginica* larvae were obtained from the East Hampton Shellfish Hatchery (East Hampton, NY, USA) within hours of fertilization. Wild broodstock were obtained from the eastern Peconic Estuary (NY, USA), which experiences normoxic and normocapnic levels of oxygen and pCO_2_ (Gobler *et al.*, 2014). Broodstock consisted of at least five individuals of both sexes to ensure genetic diversity among larvae. Broodstock were conditioned for >2 months using standard methods (Loosanoff and Davis, 1963) in seawater with a salinity of ∼28 and a pH of ∼8 through the entire gametogenic phase prior to spawning. For experiments, the following four levels of pCO_2_ were administered: a pre-industrial level (∼250 μatm pCO_2_), a modern level (∼380 μatm pCO_2_), an elevated level (∼750 μatm pCO_2_) and a high level (∼1500 μatm pCO_2_). These concentrations span the levels of pCO_2_ observed and expected in open ocean waters from the year 1750 to 2200 (Caldeira and Wickett, 2003) and can be observed in estuaries during summer (Talmage and Gobler, 2009; Cai *et al.*, 2011), when *C. virginica* spawn (Kennedy *et al.*, 1996). We note that the static pCO_2_ levels used in these experiments are likely to deviate from the diurnal, tidal and seasonal fluctuations in pCO_2_ that *C. virginica* larvae are likely to experience in estuaries (Duarte *et al.*, 2013). All treatment vessels were filled from a single reservoir containing 0.2-μm-filtered seawater obtained from eastern Shinnecock Bay to ensure equivalent seawater chemistry. Larvae were fed *Isochrysis galbana* (Tahitian strain, T-Iso), an ideal food source, at a density of 4 × 10^4^ ml^−1^ daily to maximize bivalve larval growth and survivorship through metamorphosis (Cragg, 2006; Padilla *et al.*, 2006). To promote high survivorship, all containers in contact with larvae were never exposed to chemicals or detergents. To discourage the growth of bacteria during experiments, an antibiotic solution (Sigma-Aldrich No. 4083; 5000 units of penicillin, 5 mg of streptomycin and 10 mg of neomycin per millilitre of solution) was added to each treatment vessel at 1% of its original concentration at the beginning of each experiment and at the time of each water change (approximately two times weekly). While this is not a standard practice in shellfish hatcheries, it was pursued due to the length of the experiments. This antibiotic mixture at this concentration has been shown to have no negative effects on the growth and survivorship of shellfish larvae (Padilla *et al.*, 2006).

For each experiment, larvae were distributed to each 1 l experimental vessel at a concentration of ∼400 l^−1^ in quadruplicate (*n* = 4) ∼18 h post-fertilization. Twice weekly during experiments, larvae were gently poured onto a 64 μm mesh, and the condition (live or dead) and developmental stage of each larva (veligers, pediveligers and metamorphosed) was determined visually under a dissecting microscope; every individual live larva was counted at every water change. Larvae from each vessel (*n * = 4 per treatment) were removed, counted, observed, and transferred into a new vessel with new filtered seawater, food and antibiotics within a 15 min period. Experiments were terminated after all surviving larvae in all treatments metamorphosed, after ∼3 weeks.

### Size and lipid analysis

At the end of experiments, the relative lipid content of larvae was measured as described by Talmage and Gobler (2010). Briefly, Nile Red stain was used to bind to neutral lipids and fluoresce under a fluorescein isothiocyanate filter on a fluorescence microscope (Castell and Mann, 1994; Phillips, 2002). A Nile Red stock solution was made of 1.25 mg of Nile Red crystals in 100 ml of acetone. Randomly selected larvae (*n* = 15) from each treatment were stained with a 1:9 dilution of the stock solution and 0.2-μm-filtered seawater. Larvae were exposed to the stain for ∼1.5 h, rinsed with filtered seawater, and digitally photographed with a Roper Scientific Photometrics CoolSNAP ES camera under an epiflorescence microscope to observe lipids. Photographs of each larva were taken and analysed using ImageJ^®^ software for the area of lipid accumulation, the diameter and the area of individuals. Diameters were based on shell lengths. The proportional area of lipid content was estimated as the ratio of lipid area to larval area.

### Calcification rates

In order to measure calcification rates during the veliger (day 7) and pediveliger stage (day 16), a separate set of larvae were grown at 250, 380 and 750 μatm pCO_2_ as described above (in the ‘*Larvae and treaments*’ subsection). These larvae were bubbled with the same tanked gases delivered at the same rates, held in the same vessels within the same water baths, fed the same food, with water changes on the same dates, and displayed survival rates that were similar to and not significantly different from those in the other experimental vessels at the same pCO_2_ levels. For experiments, ∼200 larvae per treatment were placed into 125 ml polyethylene bottles with 100 ml new filtered seawater; temperature was maintained at 24°C, and the bottles were continuously bubbled to deliver the same CO_2_ levels that the larvae had been grown in until that point.

Differences in the rate of calcification of larvae exposed to different levels of CO_2_ were assessed using a ^45^Ca isotope tracer method (Ho and Zubkoff, 1980). High specific activity, 9.25 × 10^6^ Bq, ^45^Ca was added to ∼100 ml of filtered seawater with larvae in four replicated, polypropylene 125 ml Nalgene bottles, resulting in a final concentration of 3.7 × 10^3^ Bq ml^−1^. A killed-control bottle was established at each level of CO_2_ via the addition of glutaraldehyde to a final concentration of 2%. After 24 h, bottles were gently gravity filtered onto 20 μm polycarbonate membranes. Time series uptake experiments were also made during the veliger stage, when sub-samples of larvae were removed at 1, 2, 4, 6 and 12 h to obtain an estimate of calcium uptake over time. All larvae retained on filters were rinsed with filtered seawater to remove surface adsorbed ^45^Ca, transferred to scintillation vials, and digested with 1 ml of concentrated HNO_3_ (15.8 n) for 1 h, after which 10 ml of UltimaGold™ scintillation cocktail was added. Beta activity of the samples was counted on a Perkin Elmer Tri-Carb 1600 liquid scintillation counter, with the discriminator window optimized for ^45^Ca detection. The weight-specific calcium uptake rate per individual was determined by the following equation:

$$\hbox{CaU} = \frac{([\hbox{dpm}]_{l} - (\hbox{dpm})_{d}] \times \hbox{Ca}_{m}}{(\hbox{dpm})_{m} \times t \times L}$$

where CaU is the calcium uptake rate (in nanograms of calcium per larva per hour), (dpm)_*l*_ is radioactivity of live larvae, (dpm)_*d*_ is the radioactivity of killed larvae, which represents non-biological factors including background, ion-exchange and isotope absorption, Ca_*m*_ is the calcium content of unit medium water (in milligrams per litre), (dpm)_*m*_ is the radioactivity of unit medium water, *t* is the length of the incubation (in days) and *L* is the number of larvae per beaker. This equation is based on the assumption that there is no significant discrimination by larvae for ^45^Ca and ^40^Ca uptake (Ho and Zubkoff, 1980). Given that bivalves exposed to high levels of CO_2_ and low levels of pH can dissolve (Green *et al.*, 2009; Talmage and Gobler, 2010), these should be considered net calcification rates. The calcium content of water was estimated by the ratio of its salinity to that of typical oceanic water of 35, which has 408 p.p.m. of Ca^2+^ (Sverdrup *et al.*, 1942).

### RNA:DNA ratios

In order to assess RNA:DNA ratios of *C. virginica* larvae, individuals were grown at the four levels of CO_2_ used in the experiments. The general experimental set-up followed the description above (see *Larvae and treatments*); precise CO_2_ levels and complete carbonate chemistry from this experiment appear in Table 1. The overall growth and RNA synthesis rate of larval shellfish was assessed by analysis of the ratio between RNA and DNA content in the organisms (Clemmesen, 1994). This approach provides an RNA:DNA ratio, which has been used in other marine organisms, especially fish larvae, to estimate growth and nutritional condition, with elevated ratios being associated with fast-growing individuals (Malzahn *et al.*, 2003, 2007). Larvae (*n * = 15) were removed from each treatment beaker (*n* = 4), poured onto a sieve, and were then macerated using a Pellet Pestle^®^ Motor, heated to 50°C for 15 min, and then frozen to −80°C for at least 90 min. Nucleic acids from pooled groups of larvae per treatment (*n* = 15) were extracted using a modified cetyl trimethylammonium bromide technique, and quantified using Quant-iT™ RiboGreen RNA^®^ and Quant-iT™ PicoGreen^®^ DNA assay kits (Invitrogen), according to the manufacturer's protocol. RiboGreen^®^ RNA and PicoGreen^®^ DNA are ultra-sensitive fluorescent nucleic acid stains for quantifying RNA and DNA, respectively, in solution. RiboGreen^®^ also binds DNA; therefore, complete DNAse digestion of the sample preceded analysis of RNA. The RNA and DNA concentrations in the extracted samples were determined by measuring fluorescence using an Applied Biosystems 7300 Real-Time PCR system-genetic analyser and compared with a standard curve of nucleic acids.

**Table 1:** Temperature, pH, carbonate chemistry, alkalinity and salinity (±1 SD) during the four-level carbon dioxide experiment

Parameter	Pre-industrial CO_2_	Ambient CO_2_	Elevated CO_2_	Elevated CO_2_
Temperature (°C)	24 ± 1.0	24 ± 1.0	24 ± 1.0	24 ± 1.0
pH	8.32 ± 0.01	8.16 ± 0.02	7.93 ± 0.001	7.68 ± 0.05
Partial pressure of CO_2_ (µatm)	249 ± 7.15	394 ± 26.7	745 ± 23.0	1430 ± 162
Saturation state of calcite	5.67 ± 0.06	4.27 ± 0.20	2.822 ± 0.06	1.69 ± 0.19
Saturation state of aragonite	3.68 ± 0.04	2.77 ± 0.13	1.83 ± 0.04	1.10 ± 0.12
Total dissolved inorganic carbon (μmol l^−1^)	1710 ± 11.8	1790 ± 31.3	1932 ± 14.2	2040 ± 11.5
CO_3_^2−^ (μmol l^−1^)	227 ± 2.45	171 ± 8.33	113 ± 2.14	67.8 ± 7.43
Total alkalinity (μmol l^−1^)	2020 ± 8.41	2040 ± 35.3	2070 ± 12.6	2090 ± 11.8
Salinity	30.0 ± 1.0	30.0 ± 1.0	30.0 ± 1.0	30.0 ± 1.0

### Scanning electron microscopy

In order to document differences in the size and structure of larval and early oysters exposed to differing levels of CO_2_, randomly chosen individuals (*n* = 6 per vessel) were mounted for scanning electron microscopic imaging as described by Talmage and Gobler (2010). Firstly, to image the outside of shells, individuals were attached at 45° relative to a level surface to a conductive substrate using carbon double-sided tape and were subsequently coated with ∼12 nm of gold using an Edwards^©^ 150B rotary pump.

In order to image the thickness and internal dimensions, cross-sections of shellfish were prepared. Individuals were mounted on glass microscope slides using ultraviolet-curing adhesive coating (Locite^©^ 4304) and were impregnated with low-vicosity epoxy (Stuers'^©^ Specifix-20) under vacuum outgassing, a step which did not alter the original shape or size of individuals. After curing, the epoxy mount was progressively ground and polished to the centreline (hinge to shell edge) of the shellfish using silicon carbide sandpapers, followed by successively finer diamond polishing grits (15, 6 and 3 μ), 0.05 μm aluminum oxide suspension and, finally, with colloidal silica. All individuals were cross-sectioned at the same location (hinge to shell edge) across the shell. Measurements of shell thicknesses were made at the precise mid-point between the hinge and valve opening on the upper and lower shell for all individuals. This mount was then attached to a conductive substrate using carbon double-sided tape and coated with ∼4 nm of gold.

Scanning electron microscopic images were collected on both types of samples with a Leo (Zeiss) Model # 1550 electron microscope using a high voltage of 20 kV and a Robinson back-scatter detector. All components of individual bivalve shells displayed in Fig. 6 were probed using an advanced energy dispersive x-ray analyzer microanalysis in the LEO (Zeiss) Model # 1550 electron microscope and were confirmed to contain almost exclusively carbon, oxygen and calcium.

### Statistical analyses

Percentage data (survival, metamorphosis) were arcsine square-root transformed prior to statistical analyses. Differences in survival, size, shell thickness, metamorphosis, RNA:DNA ratios, calcification rates and lipid indexes were assessed using one-way ANOVAs and Tukey's *post hoc* multiple comparison tests where CO_2_ levels were the treatment effects. Data that did not meet assumptions of normality and homogeneity were tested using Kruskal–Wallis tests. Statistical analyses were performed with SYSTAT 13^©^ Copyright, 2009, Systat Software, Inc.

### Allometric- and calcification-based analyses

Larvae exposed to elevated pCO_2_ levels have been shown to grow more slowly than conspecifics grown at lower pCO_2_ levels (Talmage and Gobler, 2010; Waldbusser *et al.*, 2013). As a consequence, comparing individuals in different pCO_2_ conditions at a single sampling time may make it difficult to discriminate between the direct impact of pCO_2_ on physiological performance and the indirect effects on physiology via slowed growth and development, because physiological performance is often stage and size specific. To explore this hypothesis, an allometric approach was taken whereby the length of individuals at the end of the larval stage was used as a proxy for size and age, and the shell thickness, lipid index, RNA:DNA ratio and calcification rates of individuals were divided by their length. Differences in length-based physiological performance were compared by means of a one-way ANOVA and Tukey's *post hoc* multiple comparison tests.

Given that ocean acidification compromises the ability of bivalves to calcify, it is possible that the primary impact of acidification on larvae may be compromised calcification, which has a cascading effect on the physiology of individuals. To explore this hypothesis, the length, shell thickness, lipid index and RNA:DNA ratio of individuals at the end of the larval stage were divided by their calcification rate. Differences in calcification-based physiological performance were compared by means of a one-way ANOVA and Tukey's *post hoc* multiple comparison tests.

## Results

**Figure 1:**
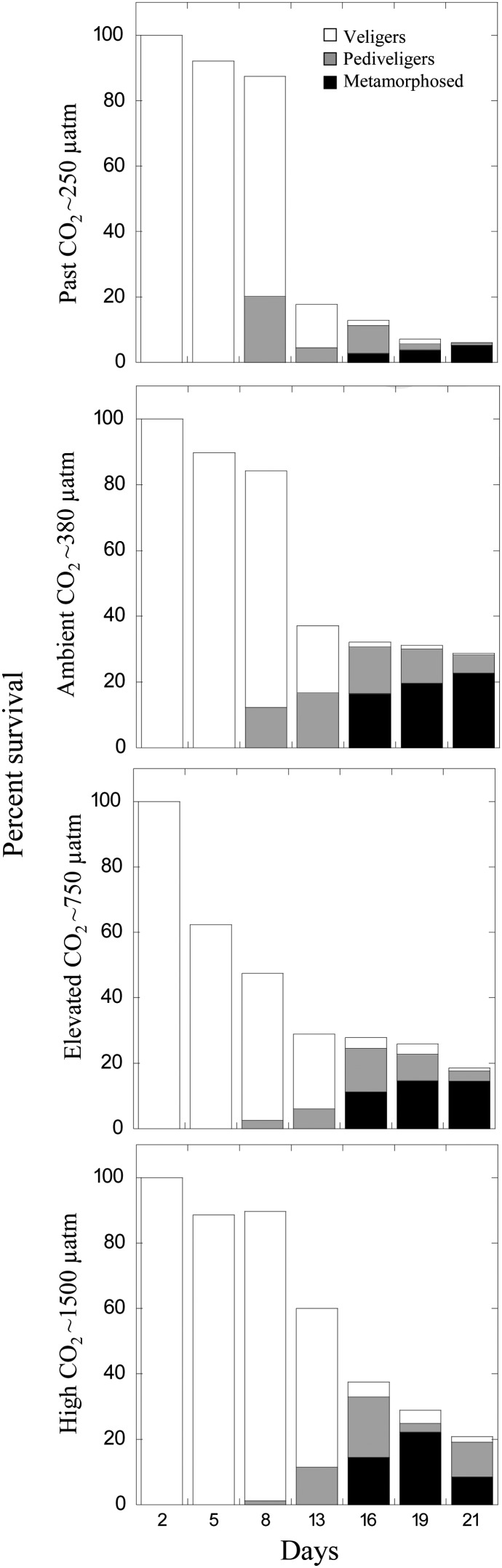
Development and survival of *Crassostrea virginica* larvae. Percentage survival and developmental stage (veliger, pediveliger and metamorphosed) of larvae grown in the presence of four levels of pCO_2_, i.e. ∼250, 380, 750 and 1500 μatm (Table 1). The relative standard deviation of larval survival among replicated vessels per treatment for all times points and experiments was 15% (*n* = 4 per treatment).

*Crassostrea virginica* larvae grown at present-day CO_2_ levels (380 μatm) generally performed better than those reared at higher and lower CO_2_ levels (250, 750 and 1500 μatm). More *C. virginica* larvae survived at present-day CO_2_ levels (29 ± 8.1%) compared with the survival at 250 (6.0 ± 3.8%), 750 (19 ± 5.5%) and 1500 μatm CO_2_ (21 ± 1.7%; Fig. 1). While survival at 250 μatm CO_2_ was significantly lower than at all other levels (*P* < 0.05; Tukey's test), the differences among the other levels were not statistically significant (*P* > 0.05).

Developmental rates were most rapid for *C. virginica* larvae grown at present-day CO_2_ levels (*P* < 0.001; Fig. 1). After 16 days of development at present-day CO_2_ levels (∼380 μatm), 51 ± 15% of surviving larvae had metamorphosed, while at ∼250, 750 and 1500 μatm, 22 ± 18, 41 ± 12 and 39 ± 0.4% had done so (Fig. 1).

**Figure 2:**
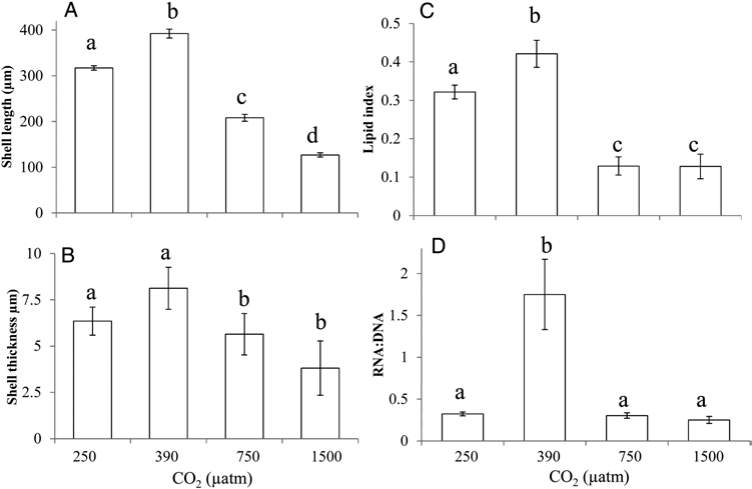
Shell lengthand thickness, lipid index and RNA:DNA ratio of *C. virginica* larvae in the presence of pCO_2_ levels of ∼250, 380, 750 and 1500 μatm. (**A**) Shell length of *C. virginica*. (**B**) Shell thickness of *C.virginica* shells at mid-point between the hinge and valve edge of the upper and lower shell of cross-sectioned individuals. (**C**) Lipid index (lipid area/total area) of *C.virginica* individuals (day 21). (**D**) RNA:DNA ratio of *C. virginica* individuals. Errorbars represent standard deviation of replicated vessels per treatment (*n* = 4 per treatment). Lower case letters signify treatments that are statistically equal (same letter) or different (different letters) as revealed via a Tukey test (*P* < 0.05).

Shell lengths of *C. virginica* larvae at day 21 were significantly larger (392 ± 9.8 μm) when grown in present-day CO_2_ conditions (380 μatm), compared with 250 μatm pCO_2_ (317 ± 4.7 μm), 750 μatm CO_2_ (208 ± 7.5 μm) or 1500 μatm pCO_2_ (127 ± 4.6 μm; *P* < 0.001; Fig. 2A). Shells of *C. virginica* larvae were also significantly thicker at present-day CO_2_ levels (8.1 ± 1.1 μm) compared with 250 (6.4 ± 0.76 μm), 750 (5.1 ± 0.53 μm) or 1500 μatm pCO_2_ (5.1 ± 0.62 μm; *P* < 0.001; Fig. 2B). The lipid content (as estimated by an index) of larvae was highest in larvae grown in 380 μatm pCO_2_ (0.40 ± 0.05) compared with the lower (0.3 ± 0.1) and the two higher levels of pCO_2_ (0.12 ± 0.01 and 0.11 ± 0.01; *P* < 0.001; Fig. 2C). The RNA:DNA ratios of larvae differed across pCO_2_ treatments. Larvae grown at 380 μatm displayed a high ratio of 1.75 ± 0.42, while larvae within other treatments had significantly lower ratios of 0.32 ± 0.02, 0.30 ± 0.03 and 0.23 ± 0.04 at 250, 750 and 1500 μatm pCO_2_, respectively (*P* < 0.001; Fig. 2D).

**Figure 3:**
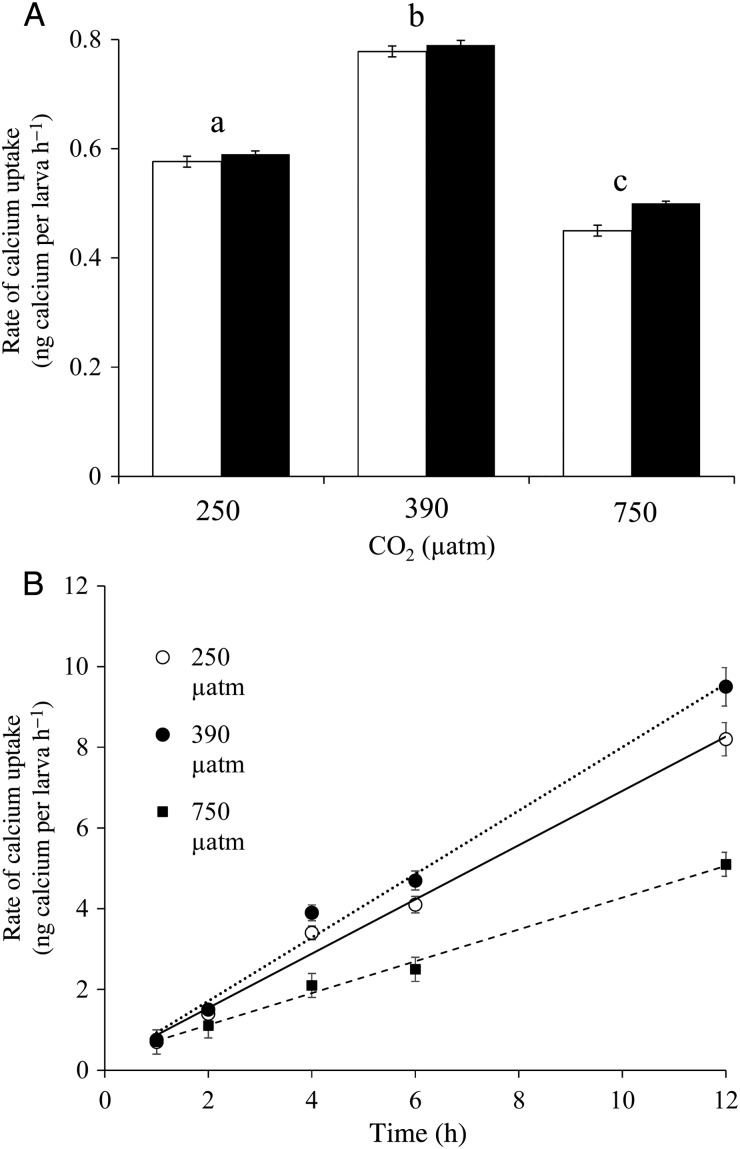
Calcification (calcium uptake) rates of *C. virginica* larvae grown under three levels of pCO_2_, ∼250, 380 and 750 μatm. (**A**) Calcification rates of day 7 veligers (filled bars) and day 16 pediveligers (open bars) during 24 h, single point measurements. (**B**) Time series measurements during a 12 h incubation with day 7 veligers with best-fit linear regressions at each CO_2_ level shown. Error bars represent standard deviation of replicated vessels per treatment (*n* = 4 per treatment). Equations and correlation coefficients of lines were as follows: 250 μatm, *y* = 0.67*x* + 0.20, *r*^2^ = 0.99; 380 μatm, *y* = 0.78*x* + 0.14, *r*^2^ = 0.99; and 750 μatm, *y* = 0.39*x* + 0.33; *r*^2^ = 0.99. Lower case letters in (**A**) signify treatments that are statistically equal (same letter) or different (different letters) as revealed via a Tukey test (*P* < 0.05).

Calcification rates of oyster larvae varied as a function of the ambient pCO_2_ levels. During 24 h measurements, calcification rates of 7-day-old veligers and 16-day-old pediveligers were significantly faster at 380 μatm CO_2_ (0.78 ± 0.003 ng calcium per larva h^−1^) compared with higher (750 μatm; 0.45 ± 0.002 ng calcium per larva h^−1^) and lower levels of pCO_2_ (250 μatm; 0.58 ± 0.003 ng calcium per larva h^−1^; *P* < 0.001; Fig. 3A). Calcification rates were highly linear during 12 h experiments with veligers, and followed trends observed during 24 h exposures, with rates being 0.77, 0.67 and 0.44 ng calcium per larva h^−1^ at 380, 250 and 750 μatm pCO_2_ (Fig. 3B).

**Figure 4:**
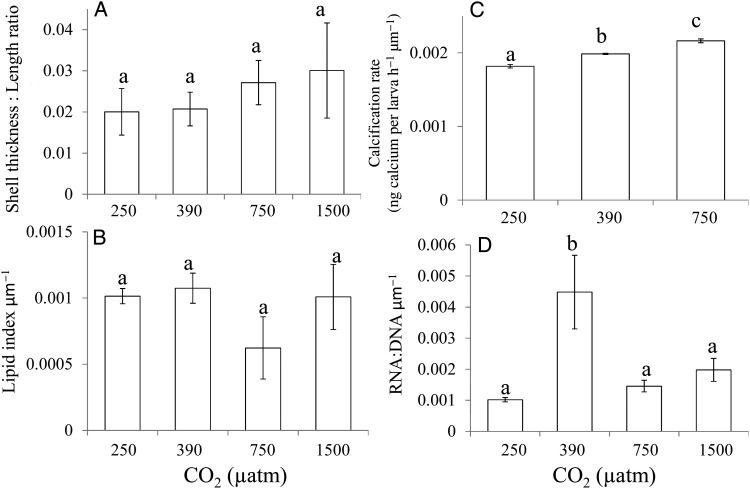
Shell length-corrected thickness, lipid index, calcification rate and RNA:DNA ratio of *C. virginica* larvae grown in pCO_2_ concentrations of ∼250, 380, 750 and 1500 μatm. (**A**) Shell thickness to shell length ratio (**B**) Lipid index per micrometre of shell length. (**C**) Calcification rate per micrometre of shell length. (**D**) RNA:DNA ratio per micrometre of shell length. Error bars represent standard deviation of replicated vessels per treatment (*n* = 4 per treatment). Lower case letters signify treatments that are statistically equal (same letter) or different (different letters) as revealed via a Tukey test (*P* < 0.05).

Scaling the oyster larval performance metrics to larval lengths indicated that some differences in larval performance may have been driven by larval size, while other attributes still differed by pCO_2_ exposure level, even when size was considered (Fig. 4). For example, there were no significant differences in the thickness or lipid index of individuals when scaled to larval length (Fig. 4A and B). Interestingly, calcification rates were slightly but significantly higher (8%) at 750 compared with 380 μatm pCO_2_ when corrected for size (*P* < 0.05; Fig. 4C). In contrast, RNA:DNA ratios of individuals grown at 380 μatm pCO_2_ remained significantly higher than those grown at higher and lower pCO_2_ levels even when corrected for the length of the larvae (*P* < 0.05; Fig. 4D).

**Figure 5:**
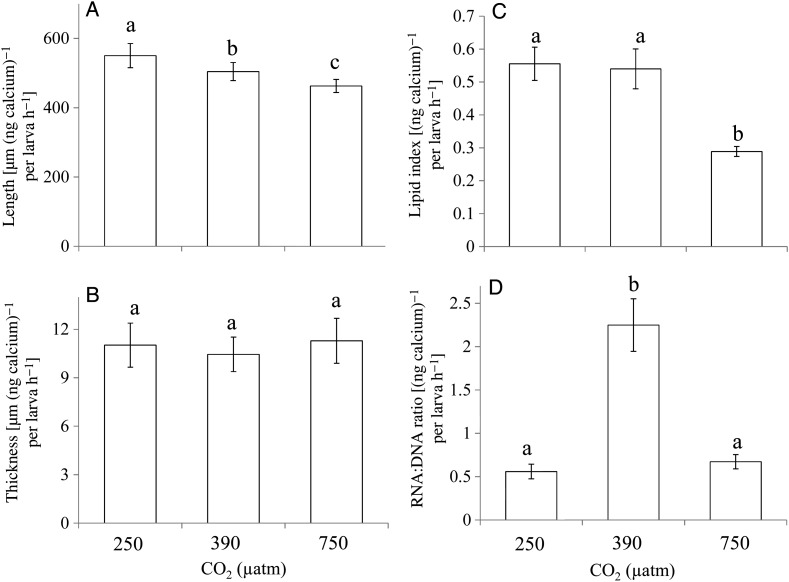
Calcification rate-corrected shell length, shell thickness, lipid index and RNA:DNA ratio of *C. virginica* larvae grown in pCO_2_ concentrations of ∼250, 380, 750 and 1500 μatm. (**A**) Micrometres of shell length per nanogram of calcium per larva per hour. (**B**) μm of shell thickness per nanogram of calcium per larva per hour. (**C**) Lipid index per nanogram of calcium per larva per hour. (**D**) RNA:DNA ratio per nanogram of calcium per larva per hour. Error bars represent standard deviation of replicated vessels per treatment (*n* = 4 per treatment). Lower case letters signify treatments that are statistically equal (same letter) or different (different letters) as revealed via a Tukey test (*P* < 0.05).

Scaling the larval oyster performance metrics to calcification rates suggested that some patterns in larval performance depended on these rates, while others were still affected by pCO_2_ exposure, even when differences in calcification rates were considered (Fig. 5). For example, once calcification rates were considered, the lengths of oyster larvae were proportional to pCO_2_ levels, with lengths decreasing as pCO_2_ levels increased (*P* < 0.05; Fig. 5A). This trend was not found with regard to shell thickness (*P* > 0.05; Fig. 5B), suggesting that this parameter was more directly affected by calcification rate. While the calcification rate-corrected lipid contents of individuals grown at 250 and 380 μatm pCO_2_ were nearly identical, the lipid content was still lower at elevated pCO_2_ even after correcting for calcification rates (*P* < 0.05; Fig. 5C). Consistent with the size-corrected RNA:DNA ratios, ratios of individuals grown at 380 μatm pCO_2_ remained significantly higher than those grown at higher and lower pCO_2_ levels, even when corrected for calcification rates (*P* < 0.05; Fig. 5D).

**Figure 6:**
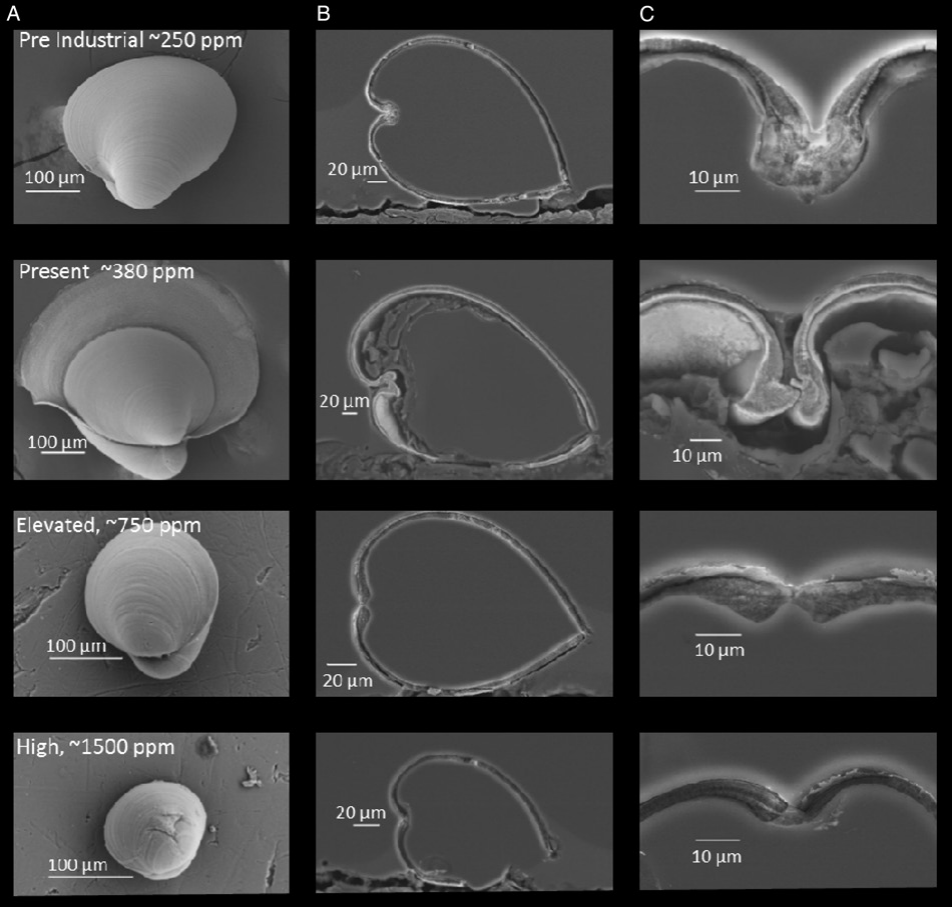
Scanning electron microscopic images of 21-day-old *Crassostrea virginica* grown in the presence of different levels of pCO_2_, i.e. ∼250, 380, 750 and 1500 μatm (Table 1). (**A**) Images of individual larvae grown at each pCO_2_ level. (**B**) Hinge-to-valve cross-sections of individuals grown at each CO_2_ level. (**C**) The hinge of individuals grown at each CO_2_ level. Note that shells of individuals at 380 μatm pCO_2_ display both the prodissoconch and dissoconch shell growth.

Scanning electron microscopic images illustrated the effects of higher and lower CO_2_ levels on shell diameter and thickness, as well as hinge structure of early life-stage oysters (Fig. 6). While there was a clear development of the metamorphic boundary between the prodissoconch and dissoconch shell parts for larvae grown in 380 μatm pCO_2_, this feature was not apparent at high and lower levels (Fig. 6). At the highest CO_2_ level in this experiment, larval shells were the smallest and thinnest and often displayed minor shell fractures (Fig. 6). Cross-sectional magnification of the *C. virginica* hinge indicated that this structure was the largest and most resembled a tongue-and-groove structure at 380 μatm pCO_2_ but appeared smaller, degraded and more disconnected at higher CO_2_ levels (Fig. 6).

## Discussion

This study demonstrated that, like many calcifying ocean animals, early life-history stages of the Eastern oysters, *C. virginica*, are negatively affected by high levels of pCO_2_. Multiple aspects of oyster physiology were strongly altered by pCO_2_, including their size, metamorphosis, lipid content, calcification rate, RNA:DNA ratios and morphology. Scaling the oyster performance to their size and calcification rates revealed that some physiological parameters may have been compromised directly by elevated pCO_2_, while others may have been controlled by slower growth or calcification rates. Unlike some other larval bivalves from North America, such as *M. mercenaria* and *A. irradians*, *C. virginica* larvae performed best at the levels of CO_2_ found in today's oceans, not lower levels (Talmage and Gobler, 2010). As discussed below, these differences may be caused by evolutionary differences among these species as well as extant physiologically driven differences in biogeography. Regardless of the causes, these findings have important implications for understanding the current and future fate of these bivalves in coastal ecosystems.

During this study, we carefully assessed the physiological condition of *C. virginica* larvae exposed to four levels of pCO_2_ by measuring metamorphosis, size, shell thickness, lipid content, RNA:DNA ratios and calcification rates, as well as the physical structure of the larval hinge. High levels of pCO_2_ (750 and 1500 μatm) were found to have a consistent, cascading negative impact on early life-stage oysters during this study. Individuals experiencing higher (750 or 1500 μatm) levels of pCO_2_ were smaller, slower to metamorphose and less calcified and had thinner shells in comparison to those at lower pCO_2_ levels. In an ecosystem setting, these smaller individuals would be more vulnerable to predation (Carriker, 1996; Tamburri and Zimmer-Faust, 1996; Marshall *et al.*, 2003). Oyster larvae grown at higher pCO_2_ (750 or 1500 μatm) also accumulated fewer lipids compared with individuals grown at lower pCO_2_ levels. Lipids are a key energy store in larvae that are used for growth during early larval stages and are accumulated in later larval stages as an energy store that is used by early juvenile stages (Waldbusser *et al.*, 2013). *Crassostrea virginica* larval growth and survival rates have been shown to parallel lipid levels (Gallager and Mann, 1986). Moreover, larvae with lower lipid contents are more likely to perish as settled juveniles (Wikfors *et al.*, 1992; Phillips, 2002; Wacker and von Elert, 2002). Given that the lipid content, size, thickness and rates of metamorphosis were all lower in individuals grown at higher CO_2_ levels, their post-metamorphosis survival rates in an ecosystem setting would probably be even lower than those found during this study. Finally, while the rates of metamorphosis of individuals reared at 380 μatm were consistent with our prior studies at this pCO_2_ level (Talmage and Gobler, 2009), the slower rates of metamorphosis at high pCO_2_ levels would leave larvae vulnerable to predation for a longer period of time in the plankton (André and Rosenberg, 1991).

Scanning electron micrographs of oysters reared at higher levels of pCO_2_ displayed a series of attributes associated with compromised calcification, including declines in the size, integrity and connectedness of their hinge, a structure that facilitates opening and closing of shells, allowing for intake of food and the excretion of waste (Eble, 2001). These compromised hinges have been observed in other bivalves exposed to high pCO_2_ (Talmage and Gobler, 2010) and may hinder the ability of individuals to obtain and process suspended particles for nutrition and thus contribute to declines in other physiological parameters, including growth and lipid content. Larvae stages of bivalves rely almost exclusively on external food sources for growth as they develop and metamorphose (Waldbusser *et al.*, 2013). Individuals grown at the highest pCO_2_ levels also displayed extremely thin and, in many cases, fractured shells that could leave individuals more vulnerable to predation (Gaylord *et al.*, 2011). Interestingly, at the lowest pCO_2_ levels, the hinge and hinge teeth were more fused than at 380 μatm, a potential indication of reduced functionality at this pCO_2_ level that may partly account for compromised performance with this treatment. While there was a clear development of the metamorphic boundary between the prodissoconch and dissoconch shell parts for larvae grown in 380 μatm CO_2_, this feature was not apparent at high and lower levels, a difference probably associated with the slower growth and delayed development of individuals exposed to higher and lower pCO_2_ levels.

At high and low pCO_2_ concentrations, oyster larvae displayed significant declines in calcium uptake and, presumably, rates of net calcification, an observation consistent with the thinner shells displayed by these individuals. Reductions in calcification are often associated with compromised shell integrity and morphology and lower growth rates of bivalves (Green *et al.*, 2009; Waldbusser et al., 2010), symptoms also seen in individuals exposed to higher pCO_2_ during this study. Calcification is a significant metabolic cost for marine organisms (Palmer, 1992). If the shells of bivalve larvae are more difficult to synthesize with increasing CO_2_ levels, this would represent an additional physiological stress that may leave less energy available for maintenance and growth. For example, juvenile oysters (*C. virginica*) exposed to elevated CO_2_ display higher metabolic rates, indicating a higher energy demand for homeostasis in these conditions (Beniash *et al.,* 2010). A similar occurrence in larvae would result in less energy being available for growth and development and, thus, could contribute to smaller sizes and other negative impacts, such as reduced lipid stores, observed during this study.

The hypothesis that reduced calcification rates have ‘trickle-down’ effects on larval physiology and performance was further supported by measurements of RNA:DNA ratios. RNA transcribes the genetic material stored in DNA and is subsequently translated by ribosomes into proteins. Hence, high levels of RNA compared with DNA (ratios >1) are indicative of an organism in an active state of transcribing RNA, synthesizing proteins and growth, while lower values are indicative of physiological impairment (Chícharo *et al.*, 1998; Malzahn *et al.,* 2003; Gobler and Talmage, 2013). While oyster larvae grown at 380 μatm had RNA:DNA ratios exceeding 1.5, those grown at higher pCO_2_ levels had values <0.5. In high concentrations of CO_2_, individuals are likely to have invested more energy in overcoming a kinetically less favourable environment for calcification. With more physiological investment in calcification, other processes, such as lipid accumulation and growth rates, were reduced, a trend also reflected in the ratios of RNA:DNA of individuals, which were also reduced at high pCO_2_, indicating that rates of transcription and growth were compromised.

Given the strong effects of pCO_2_ on calcification and, in turn, on growth of early life-stage oysters, we attempted to resolve the interacting effects of pCO_2_, calcification and size by correcting several physiological measures for size and calcification rates. This approach provided evidence of the direct effects of calcification on some attributes of oysters, the size-dependent effect on other attributes, and the overarching effect of pCO_2_ on other aspects of physiology. For example, both shell thickness and lipid content scaled with shell length, demonstrating the allometric, size-dependent nature of these attributes; pCO_2_ slowed the growth of individuals and, in turn, the smaller individuals were thinner and accumulated fewer lipids because they were less developed (Waldbusser *et al.*, 2013). Neither length nor calcification rate, however, could account for the overwhelmingly elevated RNA:DNA ratios in larvae grown at ambient pCO_2_, suggesting that these ratios are indicative of a more universally positive physiological state of early life-stage bivalves grown in the presence of ideal levels of pCO_2_ (Gobler and Talmage, 2013).

Calcification rates seemed strongly to influence some, but not all, aspects of larval oyster performance. For example, while shell thickness corrected for calcification rate did not differ among pCO_2_ levels, calcification-corrected lengths were a direct function of pCO_2_ levels, suggesting that in the most favourable conditions for calcification (lowest pCO_2_ level) the largest amounts of calcium carbonate were likely to accrete on the outer shell of larvae. The significantly lower lipid content of individuals grown at higher pCO_2_, even after calcification rates were accounted for, further affirmed that these individuals were physiologically challenged and thus unable to devote energy needed for the formation and storage of lipids compared with individuals growing in the presence of lower pCO_2_ levels (Waldbusser *et al.*, 2013).

During this study, most physiological parameters paralleled trends observed in survival, being maximal for individuals grown at ∼380 μatm, a trend not observed by Miller *et al.* (2009), who found no significant difference in the calcium content and shell size of individuals grown at 280 and 380 μatm pCO_2_. The experiments of Miller *et al.* (2009), however, began with 4-day-old larvae, while the present study exposed 18-h-old larvae to CO_2_ treatments through metamorphosis. It is during the first 48 h of oyster larval existence that calcium carbonate shells of oyster larvae are deposited for the first time (Waldbusser *et al.*, 2013), and exposure to different levels of pCO_2_ at this time can have strong effects that may be manifested days or weeks later (Barton *et al.*, 2012; Gobler and Talmage, 2013; Waldbusser *et al.*, 2013). As such, exposing 4-day-old bivalve larvae to different levels of pCO_2_ (Miller *et al.*, 2009) may mute the impacts compared with exposure during the first 2 days of life.

Ocean acidification research to date has repeatedly demonstrated that there exists substantial variability in the vulnerabilities of ocean animals to high CO_2_ (Doney *et al.*, 2009; Ries *et al.*, 2009), and bivalve larvae are not an exception (Gazeau *et al.*, 2013). A comparison of the results presented here with prior studies that have used identical methods to those employed here (Talmage and Gobler, 2009, 2010, 2011, 2012) indicate that Eastern oyster larvae are substantially less sensitive to high CO_2_ than other US east coast bivalves, such as hard clams (*M. mercenaria*) and bay scallops (*A. irradians*). For example, in the presence of moderate pCO_2_ concentrations (650 μatm), *M. mercenaria* and *A. irradians* larvae exhibited large declines (>50%) in survivorship, while *C. virginica* did not (Talmage and Gobler, 2009). Moreover, while *C. virginica* can experience depressed survival at higher levels of pCO_2_ (>1500 μatm), the relative decline in their survival is smaller than that of *M. mercenaria* and *A. irradians* (Talmage and Gobler, 2009, 2010, present study). Finally, while *M. mercenaria* and *A. irradians* experience maximal survival at low pCO_2_ concentrations (250 μatm; Talmage and Gobler, 2010), the present study has shown that the growth and survival of *C. virginica* is maximal at slightly higher pCO_2_ levels (380 μatm). Collectively, these findings demonstrate that *C. virginica* is less sensitive to acidification than some other North Atlantic bivalves and, in fact, performs best at current levels of pCO_2_ (380 μatm).

Estuaries are the principal habitat for many bivalves and are dynamic ecosystems where organisms may be exposed to a wide range of temperature, dissolved oxygen, pH and, of course, salinity. In many estuaries, and particularly in the eastern USA where *C. virginica* is abundant, freshwater tributaries are acidic and, thus, the low-salinity regions of estuaries that naturally have a lower buffering because of low alkalinity also harbour low pH and low calcium carbonate saturation states (Frankignoulle *et al.*, 1996; Salisbury *et al.*, 2008; Miller *et al.*, 2009; Hu and Cai, 2013). Bivalves are differentially suited to subsisting at low salinities, with *C. virginica* being tolerant of, and found within, lower salinities (down to 5; Kennedy *et al.*, 1996) compared with other North American bivalves, such as *M. mercenaria* (down to 18; Kraeuter and Castagna, 2001) and *A. irradians* (down to 20; Shumway and Parsons, 2006). As such, the greater resistance of *C. virginica* to acidification compared with *M. mercenaria* and *A. irradians* may partly be related to its adaptation to lower salinities and, thus, lower pH and calcium carbonate saturation states. This may also partly explain why *M. mercenaria* and *A. irradians* benefit from low CO_2_ levels (250 μatm; Talmage and Gobler, 2010) and *C. virginica* does not. The adaptation of this species to waters of lower salinity and pH makes it less likely that it would experience, and thus be adapted to, low CO_2_ levels in an ecosystem setting. Lower-salinity regions of estuaries are also likely to be associated with greater fluctuations in pH, which may foster a greater inherent genetic variability with respect to pH tolerance in these oyster populations (Curole *et al.*, 2010; Meyer and Manahan, 2010) and, thus, may also account for the greater resistance to low pH in *C. virginica*.

Molluscs first appeared during the Cambrian period ∼500 million years ago, while prehistoric oysters are likely to have evolved during the Mesozoic era (Haglund, 1998). *Crassostrea virginica* emerged as a species ∼80 million years ago (Ren *et al.*, 2010), during a period of higher levels of CO_2_ compared with the present day (Falkowski and Raven, 1997; Royer, 2006). In contrast, *M. mercenaria* and *A. irradians* have fossil records that began in the mid- to late Cenozoic (Jablonski *et al.*, 2003), which were periods of lower CO_2_ levels compared with the present day and compared with when *C. virginica* evolved (Pearson and Palmer, 2000; Pagani *et al.*, 2005; Royer, 2006; Ren *et al.*, 2010). Hence, differences in the evolution of these species may partly account for the differences in the vulnerabilities of these species to elevated CO_2_ levels. Specifically, species that evolved during periods of higher CO_2_ (i.e. *C. virginica*) may be more tolerant of higher CO_2_ levels than species that evolved during eras of lower CO_2_ levels (i.e. *M. mercenaria* and *A. irradians*) that seem to perform best at the levels of CO_2_ that were found prior to the industrial revolution. Still, open questions regarding these species, including the evolution of modern strains, the evolution of their calcification pathways and their ability to adapt to rapidly rising levels of CO_2_, remain unanswered.

Yet another reason why oyster larvae might be more tolerant to acidification could be their general population structure. Oyster reefs typically host hundreds to thousands of oysters per square metre (Kennedy *et al.*, 1996), and the microclimate around the oyster reef would be expected to be high in pCO_2_ and low in pH due metabolic processes. In contrast, other bivalves more sensitive to acidification, such as hard clams and bay scallops, do not approach these densities in the wild. Another bivalve that also exists in high-density beds, the blue mussel (*Mytilus edulis*), has also shown tolerance to acidification (Gazeau *et al.*, 2010; Thomsen *et al.*, 2010). As such, the adaption to living in dense reef communities may also contribute towards the Eastern oyster's tolerance of ocean acidification.

Regarding the optimal performance of *C. virginica* larvae in the presence of modern levels of pCO_2_, it has been postulated previously that the tolerance limits and optimal conditions of temperature and salinity for development of larvae are determined by the conditions in which gonadal development occurred prior to spawning (Davis, 1958; Newkirk *et al.*, 1977). As such, the short-term history of the parent stock could have an effect on the tolerances of the larvae. The conditioning of broodstock used during the present study was in ‘normal’ salinities and pH values, which may, therefore, have contributed to the preference of their larvae for 380 μatm pCO_2_ rather than higher or lower levels. This hypothesis should be explored in future studies.

Be it due to its evolutionary history, its biogeography, gametic conditioning, a combination of these factors or factors not considered here, *C. virginica* larvae are more tolerant of moderate pCO_2_ levels than some other bivalves. As such, this bivalve may be better suited for aquaculture and/or restoration purposes within eutrophic, acidified estuaries than bivalves common to US east coast estuaries that are more sensitive to acidification, such as *M. mercenaria* and *A. irradians*. This will also be the case in the future as atmospheric pCO_2_ levels continue to rise. Importantly, however, given that *C. virginica* can be highly susceptible to diseases and the larvae require hard substrates (e.g. oyster shell) to settle on, oyster restoration can be a challenge (Mann and Powell, 2007). Moreover, as an estuarine species *C. virginica* faces additional stressors, such as elevated temperatures and sub-optimal food, both of which have been shown additively to reduce the survival rates of bivalve larvae, including those of *C. virginica* (Talmage and Gobler, 2011, 2012). Hypoxia may also combine with acidification to threaten these bivalves (Gobler *et al.,* 2014). Lastly, all estuarine bivalves will cope with multiple acidification threats in the coming decades, namely rising levels of atmospheric CO_2_ (i.e. ocean acidification) coupled with coastal ocean acidification driven by anthropogenically enhanced organic carbon loading (Borges and Gypens, 2010; Cai *et al.*, 2011). These processes have probably contributed to the recent acidification of some estuaries (Waldbusser *et al.*, 2011) and the occurrence of pCO_2_ levels in some estuaries that are not predicted to occur in the open ocean until the end of this century but that yield lower survival rates of larvae (e.g. ≥1000 μatm; Talmage and Gobler, 2009; Cai *et al.*, 2011). As such, it seems plausible that acidification in estuaries may be contributing, at least in part, to the ‘functional extinction’ that has been observed for many oyster populations around the globe during the past century (Beck *et al.*, 2011).

## References

[1] André C Rosenberg R (1991) Adult-larval interactions in the suspension-feeding bivalves *Cerastoderma edule* and *Mya arenaria*. Mar Ecol Prog Ser 71: 227–234.

[2] Barton A Hales B Waldbusser GG Langdon C Feely RA (2012) The Pacific oyster, *Crassostrea gigas*, shows negative correlation to naturally elevated carbon dioxide levels: implications for near-term ocean acidification effects. Limnol Oceanogr 57: 698–710.

[3] Beck MW Brumbaugh RD Airoldi L Carranza A Coen LD Crawford C Defeo O Edgar GJ Hancock B Kay MC (2011) Oyster reefs at risk and recommendations for conservation, restoration, and management. Bioscience 61: 107–116.

[4] Beniash E Ivanina A Lieb NS Kurochkin I Sokolova IM (2010) Elevated level of carbon dioxide affects metabolism and shell formation in oysters *Crassostrea virginica*. Mar Ecol Prog Ser 419: 95–108.

[5] Borges AV Gypens N (2010) Carbonate chemistry in the coastal zone responds more strongly to eutrophication than to ocean acidification. Limnol Oceanogr 55: 346–353.

[6] Cai WJ Hu XP Huang WJ Murrell MC Lehrter JC Lohrenz SE Chou WC Zhai WD Hollibaugh JT Wang YC (2011) Acidification of subsurface coastal waters enhanced by eutrophication. Nat Geosci 4: 766–770.

[7] Caldeira K Wickett ME (2003) Anthropogenic carbon and ocean pH. Nature 425: 365. 14508477 10.1038/425365a

[8] Caley MJ Carr MH Hixon MA Hughes TP Jones GP Menge BA (1996) Recruitment and the local dynamics of open marine populations. Annu Rev Ecol Syst 27: 477–500.

[9] Carriker MR (1996) The shell and ligament. In Kennedy VS Newwell RIE Eble AE, eds, The Eastern oyster: *Crassostrea virginica*. Maryland Sea Grant College. University of Maryland System, College Park, MD, pp 75–168.

[10] Carriker MR Gaffney PM (1996) A catalogue of selected species of living oysters (*Ostreacea*) of the world. In Kennedy VS Newell RIE Eble AF, eds, The Eastern oyster, *Crassostrea virginica*. Maryland Sea Grant College. University of Maryland System, College Park, MD, pp 1–15.

[11] Castell LL Mann R (1994) Optimal staining of lipids in bivalve larvae with Nile Red. Aquaculture 119: 89–100.

[12] Chícharo A Chícharo L Valdés L Lopez-Jamar E Re P (1998) Estimation of starvation and diet variation of the RNA/DNA ratios in field-caught *Sardina pilchardus* larvae off the north of Spain. Mar Ecol Prog Ser 164: 273–283.

[13] Clemmesen C (1994) The effect of food availability, age or size on the RNA/DNA ratio of individually measured herring larvae: laboratory calibration. Mar Biol 118: 377–382.

[14] Cragg SM (2006) Development, physiology, behaviour, and ecology of scallop larvae. In Shumway SE Parsons GJ, eds, Scallops: Biology, Ecology, and Aquaculture. Elsevier, Philadelphia, PA, USA, pp 45–122.

[15] Curole J Meyer E Manahan DT Hedgecock D (2010) Unequal and genotype-dependent expression of mitochondrial genes in larvae of the Pacific oyster (*Crassostrea gigas*). Biol Bull 218: 122–131. 20413789 10.1086/BBLv218n2p122

[16] Davis HC (1958) Survival and growth of clam and oyster larvae at different salinities. Biol Bull 114: 296–307.

[17] Dickson AG (1993) pH buffers for sea-water media based on the total hydrogen-ion concentration scale. Deep-Sea Res I 40: 107–118.

[18] Dickson AG Sabine CL Christian JR (2007) Guide to best practices for ocean CO_2_ measurments. PICES Special Publication 3: 191.

[19] Doney SC Fabry VJ Feely RA Kleypas JA (2009) Ocean acidification: the other CO_2_ problem. Ann Rev Mar Sci 1: 169–192. 10.1146/annurev.marine.010908.163834 21141034

[20] Dove MC Sammut J (2007) Impacts of estuarine acidification on survival and growth of Sydney rock oysters *Saccostrea glomerata* (Gould 1850). J Shellfish Res 26: 519–527.

[21] Duarte CM Hendriks IE Moore TS Olsen YS Steckbauer A Ramajo L Carstensen J Trotter JA McCulloch M (2013) Is ocean acidification an open-ocean syndrome? Understanding anthropogenic impacts on seawater pH. Estuar Coasts 36: 1–16.

[22] Eble AE (2001) Anatomy and histology of *Mercenaria mercenaria*. In Kraeuter JN Castagna M, eds, Biology of the Hard Clam. Elsevier, Philadelphia, PA, USA, pp 117–216.

[23] Falkowski PG Raven JA (1997) Aquatic Photosynthesis. Princeton University Press, Princeton, NJ, USA.

[24] Feely RA Sabine CL Hernandez-Ayon JM Ianson D Hales B (2008) Evidence for upwelling of corrosive “acidified” water onto the continental shelf. Science 320: 1490–1492. 18497259 10.1126/science.1155676

[25] Frankignoulle M Bourge I Wollast R (1996) Atmospheric CO_2_ fluxes in a highly polluted estuary (the Scheldt). Limnol Oceanogr 41: 365–369.

[26] Gallager SM Mann R (1986) Growth and survival of larvae of *Mercenaria mercenaria* (L.) and *Crassostrea virginica* (Gmelin) relative to broodstock conditioning and lipid content of eggs. Aquaculture 56: 105–121.

[27] Gattuso JP Frankignoulle M Wollast R (1998) Carbon and carbonate metabolism in coastal aquatic ecosystems. Annu Rev Ecol Syst 29: 405–434.

[28] Gaylord B Hill TM Sanford E Lenz EA Jacobs LA Sato KN Russell AN Hettinger A (2011) Functional impacts of ocean acidification in an ecologically critical foundation species. J Exp Biol 214: 2586–2594. 21753053 10.1242/jeb.055939

[29] Gazeau F Quiblier C Jansen JM Gattuso JP Middelburg JJ Heip CHR (2007) Impact of elevated CO_2_ on shellfish calcification. Geophys Res Lett 34: 7.

[30] Gazeau F Gattuso J-P Dawber C Pronker AE Peene F Peene J Heip CHR Middelburg JJ (2010) Effect of ocean acidification on the early life stages of the blue mussel *Mytilus edulis*. Biogeosciences 7: 2051–2060.

[31] Gazeau F Parker LM Comeau S Gattuso J-P O'Connor WA Martin S Pörtner H-O Ross PM (2013) Impacts of ocean acidification on marine shelled molluscs. Mar Biol 8: 2207–2245.

[32] Gobler CJ Talmage SC (2013) Short-and long-term consequences of larval stage exposure to constantly and ephemerally elevated carbon dioxide for marine bivalve populations. Biogeosciences 10: 2241–2253.

[33] Gobler CJ Depasquale EL Griffith AW Baumann H (2014) Hypoxia and acidification have additive and synergistic negative effects on the growth, survival, and metamorphosis of early life stage bivalves. PLoS One 9: pe83648. 10.1371/journal.pone.0083648 PMC3885513 24416169

[34] Green MA Waldbusser GG Reilly SL Emerson K (2009) Death by dissolution: sediment saturation state as a mortality factor for juvenile bivalves. Limnol Oceanogr 54: 1037–1047.

[35] Gupta GVM Thottathil SD Balachandran KK Madhu NV Madeswaran P Nair S (2009) CO_2_ supersaturation and net heterotrophy in a tropical estuary (Cochin, India): influence of anthropogenic effect. Ecosystems 12: 1145–1157.

[36] Haglund WM (1998) Ecophenotypes of the late Cretaceous oyster *Crassostrea subtrigonalis* (Evans and Shumard, 1857), Central Alberta, Canada. In Johnston CA Haggart JW, eds, Bivalves: An Eon of Evolution. University of Calgary Press, Calgary, Alberta, Canada, pp 295–304.

[37] Havenhand JN Schlegel P (2009) Near-future levels of ocean acidification do not affect sperm motility and fertilization kinetics in the oyster *Crassostrea gigas*. Biogeosciences 6: 3009–3015.

[38] Ho MS Zubkoff PL (1980) The calcium uptake by larvae of the oyster, *Crassostrea virginica*, and the clam, *Mulinia lateralis*. Comp Biochem Physiol A Physiol 65: 143–146.

[39] Hu X Cai WJ (2013) Estuarine acidification and minimum buffer zone—a conceptual study. Geophys Res Lett 40: 5176–5181.

[40] Jablonski D Roy K Valentine JW Price RM Anderson PS (2003) The impact of the pull of the recent on the history of marine diversity. Science 300: 1133–1135. 12750517 10.1126/science.1083246

[41] Kennedy VS Newell RIE Eble AE (1996) The Eastern Oyster: *Crassostrea virginica*. Maryland Sea Grant College. University of Maryland System, College Park, MD, 734 pp.

[42] Kraeuter JN Castagna M (2001) Biology of the Hard Clam. Elsevier, Philadelphia, PA, USA, pp 228–241.

[43] Kurihara H Kato S Ishimatsu A (2007) Effects of increased seawater pCO_2_ on early development of the oyster *Crassostrea gigas*. Aquat Biol 1: 91–98.

[44] Loosanoff VL Davis HC (1963) Rearing of bivalve mollusks. Adv Mar Biol 1: 1–136.

[45] Malzahn AM Clemmesen C Rosenthal H (2003) Temperature effects on growth and nucleic acids in laboratory-reared larval coregonid fish. Mar Ecol Prog Ser 259: 285–293.

[46] Malzahn AM Aberle N Clemmesen C Boersma M (2007) Nutrient limitation of primary producers affects planktivorous fish condition. Limnol Oceanogr 52: 2062–2071.

[47] Mann R Powell EN (2007) Why oyster restoration goals in the Chesapeake Bay are not and probably cannot be achieved. J Shellfish Res 26: 905–917.

[48] Marshall DJ Bolton TF Keough MJ (2003) Offspring size affects the post-metamorphic performance of a colonial marine invertebrate. Ecology 84: 3131–3137.

[49] Matthiessen GC (2001) Early years. In Matthiessen GC, ed, Oyster Culture. Blackwell Science, Cambridge, MA, USA, pp 47–74.

[50] Meyer E Manahan DT (2010) Gene expression profiling of genetically-determined growth variation in bivalve larvae (*Crassostrea gigas*). J Exp Biol 213: 749–758. 20154190 10.1242/jeb.037242

[51] Miller AW Reynolds AC Sobrino C Riedel GF (2009) Shellfish face uncertain future in high CO_2_ world: influence of acidification on oyster larvae and growth in estuaries. PLoS One 4: pe5661. 10.1371/journal.pone.0005661 PMC2682561 19478855

[52] Newell RIE (2004) Ecosystem influences of natural and cultivated populations of suspension-feeding bivalve molluscs: a review. J Shellfish Res 23: 51–61.

[53] Newkirk GF Waugh DL Haley LE (1977) Genetics of larval tolerance to reduced salinities in two populations of oysters, *Crassostrea virginica*. J Fish Res Board Can 34: 384–387.

[54] NMFS (2010) United States National Marine Fisheries Service, Annual Commercial Landing Statistics. National Oceanic and Atmospheric Administration (NOAA).

[55] Orr JC Fabry VJ Aumont O Bopp L Doney SC Feely RA Gnanadesikan A Gruber N Ishida A Joos F (2005) Anthropogenic ocean acidification over the twenty-first century and its impact on calcifying organisms. Nature 437: 681–686. 16193043 10.1038/nature04095

[56] Padilla DK Doall MH Gobler CJ Hartson A O'Boyle K (2006) Brown tide alga, *Aureococcus anophagefferens*, can affect growth but not survivorship of *Mercenaria mercenaria* larvae. Harmful Algae 5:736–748.

[57] Paerl HW Pinckney JL Fear JM Peierls BL (1998) Ecosystem responses to internal and watershed organic matter loading: consequences for hypoxia in the eutrophying Neuse river estuary, North Carolina, USA. Mar Ecol Prog Ser 166: 17–25.

[58] Pagani M Zachos JC Freeman KH Tipple B Bohaty S (2005) Marked decline in atmospheric carbon dioxide concentrations during the Paleogene. Science 309: 600–603. 15961630 10.1126/science.1110063

[59] Palmer AR (1992) Calcification in marine mollusks: how costly is it? Proc Natl Acad Sci USA 89: 1379–1382. 11607278 10.1073/pnas.89.4.1379 PMC48454

[60] Parker LM Ross PM O'Connor WA (2010) Comparing the effect of elevated pCO_2_ and temperature on the fertilization and early development of two species of oysters. Mar Biol 157: 2435–2452.

[61] Pearson PN Palmer MR (2000) Atmospheric carbon dioxide concentrations over the past 60 million years. Nature 406: 695–699. 10963587 10.1038/35021000

[62] Phillips NE (2002) Effects of nutrition-mediated larval condition on juvenile performance in a marine mussel. Ecology 83: 2562–2574.

[63] Ren JF Liu XA Jiang F Guo XM Liu B (2010) Unusual conservation of mitochondrial gene order in *Crassostrea* oysters: evidence for recent speciation in Asia. BMC Evol Biol 10: 394. 21189147 10.1186/1471-2148-10-394 PMC3040558

[64] Ries JB Cohen AL McCorkle DC (2009) Marine calcifiers exhibit mixed responses to CO_2_-induced ocean acidification. Geology 37: 1131–1134.

[65] Roy RN Roy LN Vogel KM Porter-Moore C Pearson T Good CE Millero FJ Campbell DM (1993) The dissociation constants of carbonic acid in seawater at salinities 5 to 45 and temperatures 0 to 45°C. Mar Chem 44: 249–267.

[66] Royer DL (2006) CO_2_ forced climate thresholds during the Phanerozoic. Geochim Cosmochim Acta 70: 5665–5675.

[67] Salisbury J Green M Hunt C Campbell J (2008) Coastal acidification by rivers: a new threat to shellfish? Eos 89: 513.

[68] Schneider DW Stoeckel JA Rehmann CR Blodgett KD Sparks RE Padilla DK (2003) A developmental bottleneck in dispersing larvae: implications for spatial population dynamics. Ecol Lett 6: 352–360.

[69] Shumway SE Parsons GJ (2006) Scallops: Biology, Ecology and Aquaculture. Elsevier, Philadelphia, PA, USA.

[70] Sverdrup HU Johnson MW Fleming RH (1942) The Oceans: Their Physics, Chemistry and General Biology. Prentice-Hall, Upper Saddle River, NJ, USA.

[71] Talmage SC Gobler CJ (2009) The effects of elevated carbon dioxide concentrations on the metamorphosis, size, and survival of larval hard clams (*Mercenaria mercenaria*), bay scallops (*Argopecten irradians*), and Eastern oysters (*Crassostrea virginica*). Limnol Oceanogr 54: 2072–2080.

[72] Talmage SC Gobler CJ (2010) Effects of past, present, and future ocean carbon dioxide concentrations on the growth and survival of larval shellfish. Proc Natl Acad Sci USA 107: 17246–17251. 20855590 10.1073/pnas.0913804107 PMC2951451

[73] Talmage SC Gobler CJ (2011) Effects of elevated temperature and carbon dioxide on the growth and survival of larvae and juveniles of three species of Northwest Atlantic bivalves. PLoS One 6: pe26941. 10.1371/journal.pone.0026941 PMC3204984 22066018

[74] Talmage SC Gobler CJ (2012) Effects of CO_2_ and the harmful alga *Aureococcus anophagefferens* on growth and survival of oyster and scallop larvae. Mar Ecol Prog Ser 464: 121–134.

[75] Tamburri MN Zimmer-Faust RK (1996) Suspension feeding: basic mechanisms controlling recognition and ingestion of larvae. Limnol Oceanogr 41: 1188–1197.

[76] Thomas H Bozec Y Elkalay K de Baar HJW (2004) Enhanced open ocean storage of CO_2_ from shelf sea pumping. Science 304: 1005–1008. 15143279 10.1126/science.1095491

[77] Thomsen J Gutowska MA Saphörster J Heinemann A Trübenbach K Fietzke J Hiebenthal C Eisenhauer A Körtzinger A Wahl M Melzner F (2010) Calcifying invertebrates succeed in a naturally CO_2_ enriched coastal habitat but are threatened by high levels of future acidification. Biogeosci Discussions 7: 5119–5156.

[78] Wacker A von Elert E (2002) Strong influences of larval diet history on subsequent post-settlement growth in the freshwater mollusc *Dreissena polymorpha*. Proc Biol Sci 269: 2113–2119. 12396485 10.1098/rspb.2002.2139 PMC1691137

[79] Waldbusser GG Bergschneider H Green MA (2010) Size-dependent pH effect on calcification in post-larval hard clam *Mercenaria* spp. Mar Ecol Prog Ser 417: 171–182.

[80] Waldbusser GG Voigt EP Bergschneider H Green MA Newell RIE (2011) Biocalcification in the Eastern oyster (*Crassostrea virginica*) in relation to long-term trends in Chesapeake Bay pH. Estuar Coasts 34: 221–231.

[81] Waldbusser GG Brunner EL Haley BA Hales B Langdon CJ Prahl FG (2013) A developmental and energetic basis linking larval oyster shell formation to acidification sensitivity. Geophys Res Lett 40: 2171–2176.

[82] Welladsen HM Southgate PC Heimann K (2010) The effects of exposure to near-future levels of ocean acidification on shell characteristics of *Pinctada fucata* (Bivalvia: Pteriidae). J Molluscan Res 30: 125–130.

[83] Wikfors GH Ferris GE Smith BC (1992) The relationship between gross biochemical composition of cultured algal foods and growth of the hard clam *Mercenaria mercenaria* (L.). Aquaculture 108: 135–154.

